# Editorial: Update on diagnostic and prognostic biomarkers for women's cancers

**DOI:** 10.3389/fmed.2023.1225982

**Published:** 2023-06-14

**Authors:** Yunxia Zhu, Ming Yi

**Affiliations:** ^1^Department of Obstetrics and Gynecology, Haiyan People's Hospital, Jiaxing, China; ^2^The First Affiliated Hospital, College of Medicine, Zhejiang University, Hangzhou, China

**Keywords:** predictive biomarker, cervical cancer, ovarian cancer, uterine cancer, breast cancer, women's cancer

Women's cancers, including breast, cervical, ovarian, and uterine cancers, have a significant impact on global health, resulting in numerous fatalities and imposing substantial economic burdens on women and their families. As the world population continues to age, there is an urgent need for international efforts to reduce the incidence and mortality rates of women's cancers and improve overall women's health (Figure 1) (1, 2). The identification of novel biomarkers linked to molecular subtypes, aggressive characteristics, and prognosis is indeed crucial for the development of targeted therapies, disease monitoring, and precise treatment of women's cancers (3, 4). Advancements in imaging techniques and the utilization of biochemical biomarkers, such as proteins, DNA, mRNA, and microRNA, hold great promise as diagnostic and therapeutic tools for these types of cancers (5–8). A collection of 10 manuscripts focused on diagnostic and prognostic biomarkers for women's cancers would provide valuable insights into various aspects of these diseases. By bringing together expertise from multiple disciplines, researchers can explore different angles and approaches to advance our understanding of biomarkers in women's cancers.

**Figure 1:**
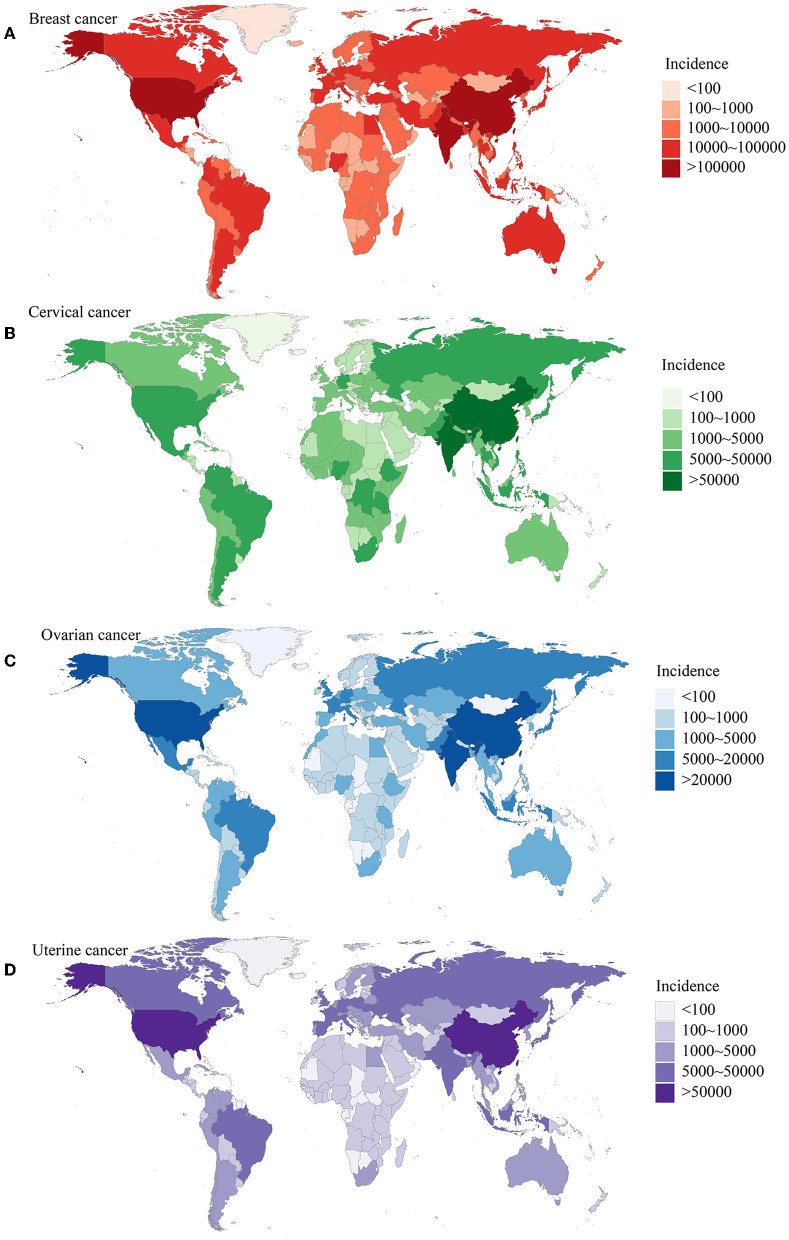
The burden of women's cancers worldwide based on the GBD 2019 database. **(A)** The incidence of breast cancer; **(B)** the incidence of cervical cancer; **(C)** the incidence of ovarian cancer; **(D)** the incidence of uterine cancer. Adapted from Yi et al. (1).

In the context of breast cancer, several biomarkers have been extensively studied for their potential as prognostic indicators (9–13). Hormone receptors, HER2, Ki-67, TP53, and BRCA1/BRCA2 are among the biomarkers that have been widely adopted for breast cancer prognosis (14, 15). These biomarkers, when combined with other clinical factors, provide a comprehensive assessment of prognosis and guide treatment decisions. However, the field of biomarker research in breast cancer continues to evolve, with ongoing exploration of novel markers and their prognostic value. The findings of Jin et al. demonstrated that circulating tumor cells (CTCs) have emerged as a valuable component in non-invasive methods for diagnosing breast cancer. Studies have shown their potential as diagnostic biomarkers, but it's important to consider the limitations and variations in the included studies when interpreting these findings. Further research and validation are necessary to establish the clinical utility of CTCs in breast cancer diagnosis. Besides, Jiao et al. found that RAI2 may play a crucial role in inhibiting the initiation and development of breast cancer. This research contributes to a better understanding of the molecular mechanisms involving RAI2 and provides potential biomarkers for predicting the prognosis of patients with breast cancer.

In the field of gynecological oncology, identifying key genes or signaling pathways has significant implications for risk stratification, early detection, personalized medicine, drug development, and understanding tumor biology (16–18). For instance, EDN3 and EREG have been identified as potential biomarkers and therapeutic targets in cervical cancer. The downregulation and hypermethylation of EDN3 suggest its involvement in cervical cancer progression, while the upregulation of EREG is associated with advanced stages of cervical cancer. Zhu et al. found EDN3 silence in cervical cancer is caused by methylation. This methylation-mediated silencing can be reversed by 5-Aza treatment in cervical cancer cells. Furthermore, EDN3 overexpression has the ability to suppress the proliferation, clone formation, and movement of cervical cancer cells. Li et al. revealed a significant association between high EREG expression and poor survival outcomes in patients with cervical cancer. Specifically, EREG expression was found to be elevated in advanced tumor stages. Enrichment analysis further demonstrated that EREG was closely associated with several important oncogenic signaling pathways. *In vitro* experiments demonstrated that knocking down EREG expression limited cell proliferation, promoted cell apoptosis, and alleviated cisplatin resistance in cervical cancer cells. Based on these findings, it can be concluded that EREG functions as a driving factor in cervical cancer progression and contributes to chemotherapy resistance. These findings provide insights into potential prognostic biomarkers and avenues for targeted therapy.

Moreover, Zhou et al. demonstrated that the impact of different HPV genotypes, multiple infections, and viral load on cervical precancerous lesions is crucial for guiding early interventions and preventing the development of cervical cancer. By considering specific genotypes, assessing multiple infections, and evaluating viral load, risk stratification can be improved, leading to appropriate management strategies for individuals with cervical precancerous lesions. In cervical cancer screening, the measurement of HPV viral load alone is not sufficient, and it should be combined with other methods to achieve maximum sensitivity and specificity. The integration of multiple detection techniques in early cervical cancer screening is essential. Therefore, further research is needed to explore the various factors that may influence the development and progression of cervical lesions. The comprehensive approach involving HPV vaccination, combined with advanced screening methods, will significantly enhance the chances of eradicating cervical cancer worldwide (19).

Apart from malignancies, this special issue also includes studies on benign gynecological tumors, such as uterine fibroids (UFs) (20). UFs are the most prevalent non-cancerous tumors that affect women of reproductive age. Traditionally, UF diagnosis has relied on transvaginal ultrasonography and pathological examination. However, in recent years, molecular biomarkers have emerged as promising tools for understanding the origin and progression of UFs. Cai et al. investigated the potential of DNA-methylated autophagy as a biomarker for UFs. Through the identification of key genes and investigation of their functional implications, they provide clinicians with a comprehensive assessment tool for UFs. The down-regulation of FOS, validated at both the transcriptional and protein levels, suggests its potential as a diagnostic biomarker for UFs. Their findings contribute to the understanding of UF pathogenesis and may guide future research efforts and clinical management strategies for this common gynecologic condition.

However, it is notable that the clinical implementation and validation of biomarkers require rigorous testing in large patient cohorts. Biomarker discovery is an ongoing process, and advancements in technologies and research will continue to contribute to the development of robust prognostic biomarkers for women's cancers.

Collectively, this Research Topic encompasses the use of biochemical biomarkers, gene expression profiling, and liquid biopsy sampling to enhance the diagnosis, treatment decision-making, and personalized therapy in breast, cervical, ovarian, and uterine cancers. By monitoring these biomarkers over time, clinicians can make informed decisions about adjusting and optimizing individualized treatment plans. These advancements hold the potential to improve patient outcomes, minimize unnecessary treatments, and facilitate long-term monitoring for these cancers.

## Author contributions

YZ and MY performed the selection of literature, drafted the manuscript, and prepared the figures. YZ collected the related references and participated in discussion. MY designed and revised the manuscript. All authors contributed to this manuscript, read, and approved the final manuscript.
